# Editorial

**Published:** 1867-02

**Authors:** 


### Editorial.

MEDICAL CONVENTION.—At the meeting of the American Medical Association, held in the City of Baltimore, May 3d, 1866, the following resolution was adopted with much unanimity, and the undersigned appointed a Committee to aid in carrying it into practical effect:—

*Resolved*, That this Association earnestly requests the Medical Colleges of the country to hold a Conventiou for the purpose of thoroughly revising the present system of Medical College instruction, and that a Committee be appointed to aid in carrying the resolution into effect.

In fulfilling the duties enjoined on them, the undersigned respectfully and earnestly invite the Trustess and Faculty of each of the regularly organized Medical Colleges in the United States to send representatives to a Convention to be held in the City of Cincinnati, Ohio, on Friday preceding the next Annual Meeting of the American Medical Association; namely, on the 3d day of May, 1867. We would also respectfully suggest that all delegates to such Convention be prepared to consider fully and act upon the following subjects:—

*First.* The adoption of a more uniform and just rate of Lecture Fees by all the Colleges in this country.

*Second.* The propriety of increasing the length of the Annual Lecture Term, and the number of Professorships.

*Third.* The adoption of measures for securing more thorough attention on the part of students, to the more elementary branches of medical science, and a more progressive order of medical studies.

*Fourth.* The practicability of requiring three Annual Courses of Lectures, instead of two, as a condition of graduation; and of making Hospital Clinical Instruction a necessary part of the Third Course.

*Fifth.* The practicability of establishing and exacting some appropriate standard of preliminary education for young men proposing to enter upon the study of medicine.

Feeling confident that a free interchange of views upon these, and such other topics as the Convention might deem proper, would result in the adoption of measures of great importance to the interests, honor, and usefulness of our profession, we again cordially and earnestly invite your coöperation.

N. S. DAVIS,<br>

S. D. GROSS,<br>

WORTHINGTON HOOKER, } Committee.<br>

M. B. WRIGHT,<br>

GEO. C. SHATTUCK,

This is a very important movement, and we sincerely hope that the invitation of the Committee will be responded to by every Medical College in the whole country. The time named, allows three working days before the opening of the next meeting of the American Medical Association, during which the important topics connected with the education of the profession could be freely and deliberately considered. It is a movement which has long been demanded by the general sentiment of the profession; and if the older Colleges in Philadelphia, New York, and Boston promptly respond to the invitation of the

Committee, the Convention will result in the accomplishment of much good.

---

Illinois State Medical Society.—The next Annual Meeting of this Society will be held on the first Tuesday in June, at Springfield. The Committees expected to report at that meeting are as follows:—

*Committee on Practical Medicine.*—Drs. J. Adams Allen, of Chicago; R. E. McVey, of Waverly; and L. T. Hewins, of Loda.

*On Surgery.*—Drs. H. W. Davis, of Paris; C. R. Parke, of Bloomington; and G. R. Bibb, of Jacksonville.

*On Obstetrics.* — Drs. DeLaskie Miller, of Chicago; N. Wright, of Chatham; J. N. Ralston of Quincy.

*On Drugs and Medicines.*—Drs. P. H. Bailhache, of Springfield; N. T. Quales, of Chicago; and J. Miner, of Waverly.

*On Ophthalmology.*—Dr. E. L. Holmes, of Chicago.

*On Spinal Curvatures and Hip-Disease.*—Drs. J. W. Freer, and R. G. Bogue, of Chicago.

*On Plastic Surgery.*—Dr. David Prince, of Jacksonville.

*On the Co-Relation of Electricity and Nervous Force.*—Dr. D. T. Kyner, of Macon.

*On Specialties and Medical Advertising.*—Drs. T. D. Fitch and N. S. Davis, of Chicago; and D. Prince, of Jacksonville.

*On the Radical Cure of Reducible Hernia.*—Dr. G. T. Allen, of Springfield.

*On Syphilis.*—Dr. W. M. Chambers, of Charleston.

We hope the profession in every part of the State will be represented, for we are sure the meeting will be both interesting and profitable. The Volume of Transactions for 1866, has been published and copies sent to all members who are credited with the payment of the annual assessment on the books of the Treasurer.

---

Rush Medical College Commencement.— The twenty-fourth Annual Commencement of this Institution was held on Wednesday evening, January 30th, 1867; at the end of their usual 16 weeks Lecture Term. Prof. J. V. Z. Blaney, who had been lately elected President of the Faculty, in the place of the late Prof. Brainard, conferred the degree of M.D. on 72 candidates; being about 20 less than the number graduated the year previous. Before conferring the degrees, Prof. Blaney paid an appropriate tribute to the memory of his predecessor, Prof. Brainard.

The Valedictory Address was delivered by Prof. E. Ingalls, and consisted mainly, as it should, of plain, practical, common sense instructions to the graduating class.

It was officially announced that Prof. Moses Gunn, of Detroit, had received and accepted the appointment to the chair of Surgery, made vacant by the death of Prof. Brainard.

---

Chicago Medical College Commencement.—The Eighth Annual Commencement Exercises of this Institution will take place on Tuesday evening, March 5th, 1867; and will be preceded by a public examination of the candidates in class, during the preceding day. There is an excellent class in attendance, and we have no doubt but its members will sustain themselves creditably, throughout the unusually extended examinations to which they are subjected in this College.

---

Physician's Pocket Record.—We have received, from the office of the *Philadelphia Medical and Surgical Reporter*, a copy of the *Pocket Record*, which was noticed and its contents quoted in a recent number of the Examiner. It is published and bound in excellent style, and in its size, arrangement, and contents is, perhaps, the most convenient and useful visiting list and memorandum book for the physician that has yet been published. Price $1.50.

---

Books not Received.—We see, by our exchanges, that Dr. D. Prince, of Jacksonville, has published a volume on *Orthopædic Surgery*, which is a revision of his report on that subject to the Illinois State Medical Society. Though the publishers have not favored us with a copy, we nevertheless assure our readers that it is one of the best practical works on that subject that has been written, either in this country or in Europe.

We also notice, in some of our exchanges, that a new edition of *Wood's Practice of Medicine* has been issued, carefully revised by its author. Though our recommendation of the work has, probably, aided in its sale throughout the North-west during the last 15 years, more than any other one individual, yet we were not favored with a copy of the former editions, nor, thus far, one of this. We do not complain of this, for, happily, we are able to buy the new edition, and independent enough to recommend it, as we have done the former ones, so far as we deem it worthy. And if the publishers should send us a dozen copies, we would do nothing more.

---

### NOTES UPON GENERAL SCIENCE.

Geological Discoveries in the Excavation of Chicago Tunnel.—In order to obtain for the City of Chicago a full supply of pure lake water, a tunnel has been dug, commencing at the shore and extending two miles outward, under the bed of Lake Michigan. This excavation is, its whole distance, through the formation called by geologists, "glacial drift," "boulder formation," etc. It is a hard clay, full of boulders, many of which show, by peculiar marks and parallel scratches on their surfaces, that they have, at some former period, been imbedded in moving glaciers, like those of the Alps. Prof. Agassiz maintains that wherever this drift is found, it has been deposited, not by water, but by glaciers. Other geologists dispute this, and claim that the drift was deposited by water, and that the boulders were dropped from icebergs floating on the surface. A very important test question in the case was, whether the glacial drift was stratified or not. If stratified, it is a water deposit. If not, it is a glacier deposit. The excavations in the tunnel show that the glacial drift at this locality is clearly stratified, and, therefore, a water deposit.

In excavating, the workmen opened a spring of inflammable gas, which burned four or five days and then ceased.

A very curious discovery was made, of numerous "pockets" or masses of clean, loose gravel, bedded in the clay in such forms and singular positions as to show that they were originally masses of frozen gravel, dropped, like boulders, to the bottom, and bedded in the accumulating clay, and, finally, thawed out by internal heat after they were covered up in the earth.

Death of Major Robert Kennicott.—This distinguished young naturalist went on a scientific expedition to the region near Behring's Straits in connection with the officers of the Overland Telegraph Line, which is to enter Asia by that route. He expected to make very valuable discoveries and collections for the Chicago Academy of Sciences He died instantaneously, on the banks of the Youkan River, in the Russian Possessions, probably from apoplexy. His remains are on the way home. He is lamented as the most active and enthusiastic naturalist of the West, and his death is a great loss to American science.

Fossil Remains of the Island of Malta.—Investigations made in the ravines and caverns of Malta, show an astonishing number of elephants, hippopotami, and other large animals, to have lived there at a former age. The appearances indicate that crowds of animals died at once, from some great convulsion of nature. The geologists who have investigated the deposits, are of the opinion that more animals died at one time than the whole island could support in its present size. They believe that Malta must, at a former period, have been connected with Africa by a tract of territory which is now beneath the sea; and that it became submerged by some convulsion which destroyed the land animals. If this extensive tract were lowest near the present African shore, or began to sink first near there, the effect would be to drive all the animals before the advancing waters until they were concentrated in vast numbers upon the high grounds of the present island, there to die by starvation, or by the submergence of their last refuge in the waves.

---

The expenditures of the New York State Inebriate Asylum from its organization to September 1, 1866, according to a recent report, have been $401,635 29.

Mortality for the Month of January.—The subjoined is the mortality report of Health Officer Bridges for the past month. It includes an accurate list of the number of deaths, with places of nativity, cause of death, a comparative statement, and other valuable information:—

CAUSES OF DEATH.

| | | | |
|---|---|---|---|
| Accidents, | 3 | Fever, Remittent, | 3 |
| Bronchitis, | 1 | Fever Purperal, | 1 |
| Burned, | 1 | Fever, Scarlet, | 21 |
| Cancer, | 2 | Fever, Spotted, | 1 |
| Childbed, | 8 | Fever, Typhoid, | 11 |
| Colic, | 1 | Fever, Congestive, | 1 |
| Congestion of Brain, | 3 | Hydrocephelus, | 3 |
| Congestion of Lungs, | 2 | Inflammation of Brain, | 3 |
| Consumption, | 40 | Inflammation of Lungs, | 10 |
| Convulsions, | 38 | Inflammation of Bowels, | 3 |
| Croup, | 11 | Inflammation not stated, | 3 |
| Cold, | 1 | Killed, | 1 |
| Diabetes, | 1 | Old Age, | 11 |
| Disease of Brain, | 1 | Pneumonia, | 1 |
| Disease of Bowels, | 1 | Poisoning, | 1 |
| Disease of Heart, | 6 | Phthisis Pulmonalis, | 2 |
| Disease of Kidneys, | 4 | Rheumatism, | 1 |
| Disease of Liver, | 2 | Stillborn, | 22 |
| Disease of Lungs, | 4 | Scald, | 1 |
| Disease of Spine, | 2 | Small-Pox, | 1 |
| Diarrhœa, | 4 | Suicide, | 2 |
| Diphtheria, | 8 | Teething, | 12 |
| Delirium Tremens, | 2 | Whooping-Cough, | 10 |
| Dropsy, | 5 | Unknown, | 20 |
| Erysipelas, | 2 | | |
| Epilepsy, | 1 | Total, | 299 |

Ages of the Deceased.—Under 5 years, 157; over 5 and under 10 years, 15; over 10 and under 20, 11; over 20 and under 30, 28; over 30 and under 40, 32; over 40 and under 50, 22; over 50 and under 60, 6; over 60 and under 70, 12; over 70 and under 80, 6; over 80 and under 91, 1; unknown, 9. Total, 299.

NATIVITIES.

| | | | | | |
|---|---|---|---|---|---|
| Chicago, | 156 | Germany, | 44 | On the Sea, | 1 |
| Other States, | 39 | Ireland, | 28 | Unknown, | 5 |
| Canada, | 5 | Norway, | 3 | | |
| Denmark, | 1 | Poland, | 1 | Total, | 229 |
| England, | 12 | Scotland, | 4 | | |

DIVISIONS OF THE CITY.

North,......... 93 | South,.......... 87 | West,.........119 | Total,......... 299

Total number during the month of December, 309

Total number last year for the month of January, 292

---

Metallic Spectacles.—M. Foucault recently communicated to the French Academy of Sciences the fact that the sun may be viewed through a lens covered with silver leaf. The sun's disc, shorn of its beams, can thus be clearly seen. Subsequently, M. Melscius made a useful application of Foucault's discovery. Having been injured while making an experiment in the laboratory, his eyes were painfully affected by light. In this condition he had recourse to spectacles with black glasses, such as are used by engine-drivers; over these he put green glasses, which answered pretty well; but on further experiment, he found the best method was to use pale-blue goggles, covered with silver or gold film, and these he recommends to all persons troubled with weak eyes.

---

THE HEADQUARTERS OF DRUNKENNESS.—Liverpool has been pronounced the most drunken town in England. And it is true. Its extreme drunkenness arrests the attention of the judges, its pauperism weighs heavily upon the rate-payers; its rate, fifty-six per thousand, appalling. The drunken cases dealt summarily with by the magistrate are set down at the annual rate of one in thirty-three of the population. The habitual drunkards, in their periodic appearances before the bench, form an endless chain of besotted creatures. According to the recently published judicial statistics there are 3,100 habitual drunkards in Liverpool, and they are about equally divided as to sexes.—*Liverpool Albion.*

---

POISONED BREAD AT WINONA, ILLINOIS.—It will be remembered that in July last, at a hotel in Winona, Illinois, a large number of persons were poisoned by eating warm biscuit at breakfast. The case attracted much attention, from the fact, that a few days before, some forty persons were in a similar manner poisoned at a hotel in Indianapolis; and from the circumstance, that self-raising flour was charged with having produced the poisoning at Winona. The public had not forgotten the bread-poisoning on a large scale in the State of N. Y., where the metallic lead used to bind the burr-blooks composing the mill-stones had been abraded and mixed with the flour in grinding. The investigation of the Indianapolis case by Prof. Wormley, of Ohio, revealed in the sour milk employed, with salæratus, to make the bread light, five and a-half grains of tartar emetic and a trace of arsenic, in a single pint. A similar case at Atlanta, Georgia, in which a whole family was poisoned, was traced to arsenic introduced by a servant.

The Winona case was taken up by the physicians and druggists of the place, and prosecuted till they and the victims generaly became satisfied that the self-raising flour had nothing to do with the poisoning. The investigation was renewed by

Prof. Horsford, of Cambridge, Mass., and the results at which he arrived are embodied in the following statement:—

1. That there was substantially no self-raising flour in the batch from which the biscuit were made.

2. That poison was introduced into the biscuit, through the sour milk employed with salæratus, in ordinary flour, to make the biscuit light.

3. That the poison was introduced by design.

4. That the poison was arsenic.—*Medical and Surgical Reporter.*

---

A Remarkable Solvent.—It is now discovered, it appears, that if a piece of copper be dissolved in ammonia, a solvent will be obtained, not only for lignine, the most important principle of all woody fibre — such as cotton flax, paper, etc.—but also for substances derived from the animal kingdom, such as wool and silk. By the solution of any of these an excellent cement and water-proofer is said to be formed; and, what is equally important, if cotton fabrics be saturated with the solution of wool, they will be enabled to take the dyes — such as the lac dye and cochineal hitherto suited to woollen goods only. —*Exchange.*

---

A New Instrument for Subcutaneous Injections.—M. Bouillaud lately presented to the Academy of Medicine of Paris an invention of M. Dancet, consisting of a hollow needle adapted to a metallic tube, ending in a small cup covered with an india-rubber membrane. By slight pressure upon the latter the fluid is injected into the areolar tissue, and a simple mechanism within the cup allows of the counting of the drops injected. Another and simpler needle on the same principle may be used for vaccination.—*Buffalo Med. and Sug. Journal.*

---

White Paste.—Make the following mixture, which will adhere to any substance:—Sugar of lead and alum, each 720 grs.; both are dissolved in water. Take 2½ ounces of gum arabic, and dissolved in two quarts of warm water. Mix in a dish 1 pound of wheat flour with the gum water cold, till pasty inconsistence. Put the dish on the fire, and pour into it the mixture of alum and sugar of lead. Shake well, and take it off the fire when it shows signs of ebullition. Let the whole cool, and the paste is made. If the paste is too thick, add to it some gum water, till in proper consistence.—*Journal of Applied Chemistry.*

Cholera and Quarantine.—The *British Medical Journal* says, that it is the intention of the Egyptian government to institute precautionary measures against the importation of cholera by the Mohammedan pilgrims next year. The quarantine measures which it has been proposed should be adopted, have been framed with regard to both vessels and caravans, and are to the following effect:—All vessels with pilgrims are to be subjected to interrogation, and if found to have had cholera on board, are to be sent to perform quarantine. All caravans are likewise, if necessary, to undergo quarantine, for which special accommodation is proposed to be provided. And should cholera break out in the Hedjaz, it is proposed that no communication between that province and Egypt should be allowed by sea.—*Philadelphia Med. and Surg. Reporter.*

---

The Gain in the Average Duration of Human Life.—Dr. C. A. Logan, in his "Report on the Sanitary Relations of the State of Kansas," cites the example of Geneva, in Switzerland, where an accurate record of the population, births, and deaths, has been kept for more than three centuries past, or since the year 1560. By a series of historical and statistical compilations, M. Mallet has ascertained that from the year 1560 to the year 1600, the mean duration of the lives of the people was, in round numbers, twenty-one years and two months. During the seventeenth century, the mean life had increased to twenty-five years and nine months; and in 1833, it had reached forty-five years and five months, being nearly double what it was about two centuries before. This result was brought about by a most salutary regulation of the public health, through which much of the former unnecessary sickness was prevented.

---

The Paris Exposition.—"The improvements in Hospital construction and equipment, in surgical appliances, in means of transportation of sick and wounded, etc., resulting from the vast experience of the War, are considered worthy of exhibition as an evidence of National progress, and with this view, models of U. S. General Hospitals, with their equipment, of ambulances, litters, medicine wagons, etc., have been prepared, and will be forwarded through the proper channels as the contribution of the Medical Department U. S. A., to the Paris Exposition."

---

International Medical Congress of Paris.—An International Medical Congress is to be held in Paris on the 16th of

August, 1867, under the auspices of his Excellency the Minister of Public Instruction. The Congress will be exclusively scientific, and will last two weeks. The labors of the Congress will include communications upon questions proposed by the committee, and also upon subjects not in their programme, which runs as follows:—1. The Anatomy and Pathological Physiology of Tubercle—On Tuberculization in different Countries, and its influence on the General Mortality. 2. The general Accidents which cause Death after Surgical Operations. 3. Is it possible to propose to the various Governments efficacious measures for restraining the Propagation of Venereal Diseases? 4. On the influence of the Dietary of different Countries in the Production of given Diseases. 5. On the influence of Climate, Race, and different Conditions of Life on Menstruation in various Countries. 6. On Acclimatization of European Races in Tropical Countries. 7. On the Entozoa and Entophytes which may be developed in Man.

Those who desire to bring forward communications on these or any other subjects, are requested to address their manuscripts to the General Secretary at least three week (July 26th) before the opening of the Congress.

With a view of limiting and defining the questions in the programme, the committee has appended to each article commentaries, which we cannot now quote, but to which we shall subsequently refer, indicating the points to which it desires that papers should be especially directed. Foreigners may become members of the Congress by addressing a communication to Dr. Jaccoud, Secretaire General, Rue Drouot 4, á Paris.—*London Lancet.*

The idea of this Convention is an excellent one, and steps have been taken in Boston to secure a representative from members of the Massachusetts Medical Society residing within the Suffolk District.—*Boston Medical and Surgical Journal.*

---

EUSTACHIAN TUBE NORMALLY CLOSED EXCEPT IN DEGLUTITION.—Dr. James Jago, in a communication on the Functions of the Tympanum, published in the *British and Foreign Medico-Chirurgical Review*, defends with much plausibility the view which he had propounded before, in an Essay on the Eustachian Tube, that the normal condition of this passage is that of closure, except during the act of deglutition. This opinion is based on experiments in his own person, aided by an accidental condition of the fauces, arising from contraction of the tissues on the right side, following amputation of a portion of the uvula. His paper is a very interesting one, and his arguments are most convincing. With regard to the provision for opening the tubes on occasion, he says:—

"That it is not the egress of sonorous vibrations from the tympanum which is to be feared, that being a matter of indifference.

"But there must be a provision against the ingress of aërial undulations from the throat, which, if admitted, would threaten the membrana tympani with incessant oscillations, and endanger both its integrity and that of the complex and delicate apparatus in connection, and violate the peace of the labyrinth *via* this sudden route with all the sonorous impulses impressed upon the animal's breath.

"That, therefore, the moment seized for bringing the tympanum into communication with the fauces must be one in which there can be no respiratory current.

"That the only instant compelling a suspension of respiration is that in which the act of swallowing is performed, and must therefore be embraced for the service just named.

"Finally, the same rule secures the tympanum against the introduction of gastric gases, etc., evolved through the fauces." —*Ibid.*

---

Prof. Unger, the eminent Viennese botanist and palæontologist, has been recently examining the bricks used by the ancient Egyptians in the construction of the Pyramids, and more particularly those of the Pyramid of Dashour. He has discovered that the mud of which they were made contained not only a quantity of animal and vegetable matter, but also fragments of many manufactured substances, leading to the conclusion that Egypt enjoned a high degree of civilization upward of 5000 years ago.

---

Insufflation of Medicated Powders in Gleet.—Dr. Mallez has contrived an instrument by which medicated powders can be conveyed to the membranous portion of the urethra, a region where lies the cause of the gleet, and which region ordinary injections cannot reach. The instrument is chiefly composed of a catheter opened at both ends, another which glides into it, and an apparatus for receiving and projecting the powders. M. Ricord presented the instrument to the Academy of Medicine of Paris, and cases are mentioned in which excellent results were obtained by insufflating subnitrate of bismuth. It is plain that, by previous micturition, the powder may remain hours in the passage; and it is thought that the vagina, uterus, or any fistuluous tract may be reached in the same way.—*Lancet.*

---

Rapidity of Nerve Action.—Haller attempted in reading the Æneid aloud, to count the number of letters he could pronounce in a minute. Finding that he could pronounce 1,500, among which the R, according to his statement, requires ten successive contractions of the stylo-glossus, he affirms that a muscle can contract and relax itself 15,000 times in a minute; and as the time of relaxation is as long as that of contraction, each contraction requires about 1-30,000 of a minute, or 1-500 of a second. From this, Haller concludes that the nervous agent requires the 1-500 of a second to go from the brain to the stylo-glossus muscle.—*Revue des Cours Scient.*

